# Application of Compact Folded-Arms Square Open-Loop Resonator to Bandpass Filter Design

**DOI:** 10.3390/mi14020320

**Published:** 2023-01-26

**Authors:** Augustine O. Nwajana, Emenike Raymond Obi

**Affiliations:** 1 School of Engineering, Medway Campus, University of Greenwich, Chatham ME4 4TB, UK; 2 Raysoft AssetAnalytics, Regina, SK S4N 7S1, Canada

**Keywords:** resonator, half-wavelength, square open-loop, folded-arms square open-loop, bandpass filter, coupling, microstrip

## Abstract

Folded-arms square open-loop resonator (FASOLR) is a variant of the conventional microstrip square open-loop resonator (SOLR) that facilitates further device size miniaturization by having the two arms of the conventional SOLR folded inwards. This paper highlights the benefits of this brand of compact SOLR by implementing a five-pole Chebyshev bandpass filter (BPF) using compact FASOLR. The test BPF is presented, with centre frequency of 2.2 GHz, fractional bandwidth of 10%, passband ripple of 0.04321 dB, and return loss of 20 dB. The design is implemented on a Rogers RT/Duroid 6010LM substrate with a dielectric constant of 10.7 and thickness of 1.27 mm. The filter device is manufactured and characterised, with the experimentation results being used to justify the simulation results. The presented measurement and electromagnetic (EM) simulation results demonstrate a good match. The EM simulation responses achieve a minimum insertion loss of 0.8 dB and a very good channel return loss of 22.6 dB. The measurement results, on the other hand, show a minimum insertion loss of 0.9 dB and a return loss of better than 19.2 dB. The filter component has a footprint of 36.08 mm by 6.74 mm (that is, 0.26 λg × 0.05 λg), with λg indicating the guided wavelength for the 50 Ohm microstrip line impedance at the centre frequency of the proposed fifth-order bandpass filter.

## 1. Introduction

**Figure 1:**
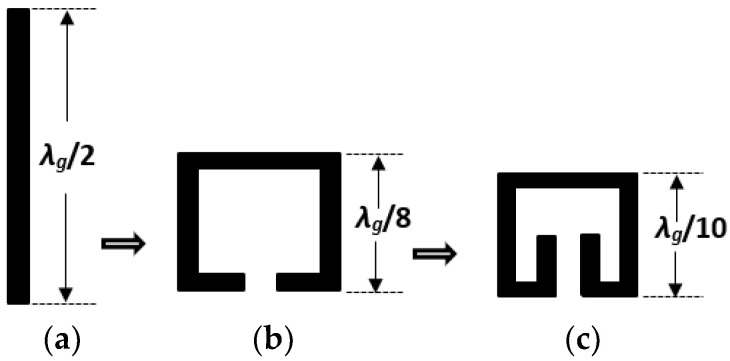
Microstrip resonator structure evolution: (**a**) Half-wavelength structure; (**b**) Square open-loop structure; (**c**) Folded-arms square open-loop structure.

Conventional microstrip square open-loop resonators (SOLRs) evolved from the well-known half-wavelength resonator shown in Figure 1a. SOLRs are attained by bending half wavelength resonators to form a square shape, while leaving a small gap, as demonstrated in Figure 1b. The SOLR is a very popular structure that finds widespread applications in filter design. This is due to its small size of approximately 1/8 of a quarter-wavelength along each side of the square [1]. The popularity and versatility of the SOLR in filter design means that numerous researchers have used it in implementing their designed filtering components [2,3,4,5,6,7,8,9,10,11,12].

The modern-day electromagnetic (EM) spectrum is becoming overcrowded and is heavily crammed with a variety of wireless signals and other communication and sensing circuits and components. EM waves of frequencies, varying from 300 MHz up to 300 GHz, remain classified as microwaves. This frequency band matches the free space wavelengths of 1 m to 1 mm, in that order. EM waves of frequencies varying from 30 GHz to 300 GHz stay classified as millimetre-waves due to their wavelengths that fall directly over 1 mm and directly under 10 mm. The radiofrequency (RF) band falls somewhere beneath the microwave range, though the boundary that separates the radio frequency and microwave bands is subjective and changes based on the method established for developing the band. Due to the overcrowding of modern radio frequency and microwave communication systems, researchers in this field have continuously worked on miniaturizing front-end components to ensure compact size and improved performance. The compact folded-arms square open-loop resonator (FASOLR) presented in this paper is one such research efforts aimed to further reduce the footprint of communication systems components. Microstrip folded-arms square open-loop resonators [13,14,15] are achieved by having the two arms of the conventional SOLR [16] folded inwards, as shown in Figure 1c. This leads to a further reduction of approximately 20% when compared to the conventional SOLR, which is already popular for its compactness. Though the coupling scheme of the FASOLR is like that of the SOLR reported in [16], the FASOLR has improved the physical device footprint by about a 20% reduction. The length of each side of the FASOLR is measured about 1/10 of a guided wavelength, as indicated in Figure 1c, while that of the SOLR is about 1/8 of the guided wavelength, as reported in [16] and shown in Figure 1b. The coupling between the two folded arms of the FASOLR ensures that the size reduction is greater than the 20% expected based on the resonator physical dimension [14].

Bandpass filters have been extensively researched and described in the literature [17,18,19,20,21,22,23]. This type of filter has wide applications in wireless communications, particularly in wireless transmitters and receivers [19]. In the transmitter, this type of filter is used to restrict the bandwidth of the output signal to the allocated channel for transmission. In the receiver, conversely, bandpass filters are used to ensure that only the desired frequencies are allowed at the receiving end of the wireless communication system. Most design requirements of bandpass filters, such as selectivity, cost, size, sensitivity to environmental effects, power handling capacity, and in-band and out-of-band performance metrics, are critical specifications when it comes to the development of radio frequency (RF) and microwave communication front ends. Filter design engineers and technicians are often required to make compromises between numerous conflicting requirements, as it is rather difficult or even physically and/or electrically impossible to simultaneously achieve all design criteria and requirements. For example, achieving higher channel selectivity usually requires the use of more resonators, which will then result in higher insertion loss along the transmission path [1]. The filter reported in this paper is compact, with very good performances including low return and insertion losses, high selectivity, and very sharp roll-off.

## 2. Circuit Modelling of Bandpass Filter

**Figure 2:**
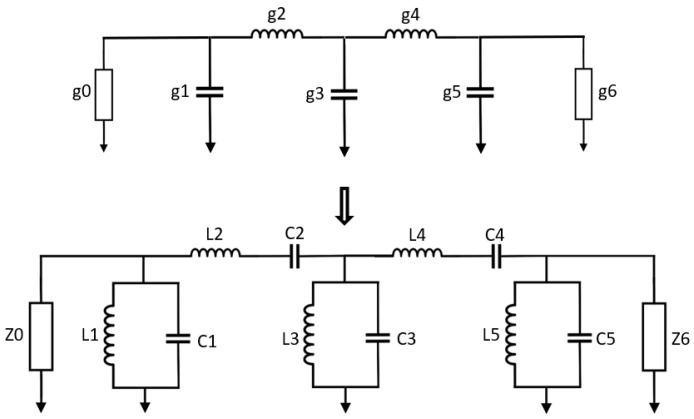
Fifth-order bandpass filter transformation (*g*_0_ = *g*_6_ = 1.0, *g*_1_ = *g*_5_ = 0.9714, *g*_2_ = *g*_4_ = 1.3721, *g*_3_ = 1.8014; Z_0_ = Z_6_ = 50 Ω, *L*_1_ = *L*_5_ = 0.3724 nH, *L*_2_ = *L*_4_ = 49.6310 nH, *L*_3_ = 0.2008 nH; *C*_1_ = *C*_5_ = 14.0548 pF, *C*_2_ = *C*_4_ = 0.1054 pF, *C*_3_ = 26.0638 pF).

A fifth-order BPF circuit has been chosen as a case study in this paper. The circuit model is formed by transforming a model normalized fifth-order Chebyshev lowpass filter prototype into series and parallel LC resonators, as demonstrated in Figure 2. The conversion relies on Equations (1) and (2) [1]; *g*_1_, *g*_2_, *g*_3_, *g*_4_ and *g*_5_ are the filter parameters; Z_0_ and Z_6_ are the characteristics impedances at the input/output terminals; *C*_1_, *C*_2_, *C*_3_, *C*_4_ and *C*_5_ are the calculated capacitance values; *L*_1_, *L*_2_, *L*_3_, *L*_4_ and *L*_5_ are the calculated inductance values. The intended filter is composed with a center frequency, *f*_0_ = 2.2 GHz, a fractional bandwidth, *FBW* = 10%, a 0.04321 dB passband ripple, and a 20 dB passband return loss.

(1) Cp=gpω0Z0FBW ; Lp=Z0FBWgpω0 ; p→odd numbers

(2) Cs=FBWgsω0Z0 ; Ls=gsZ0ω0FBW ; s→even numbers

**Figure 3:**
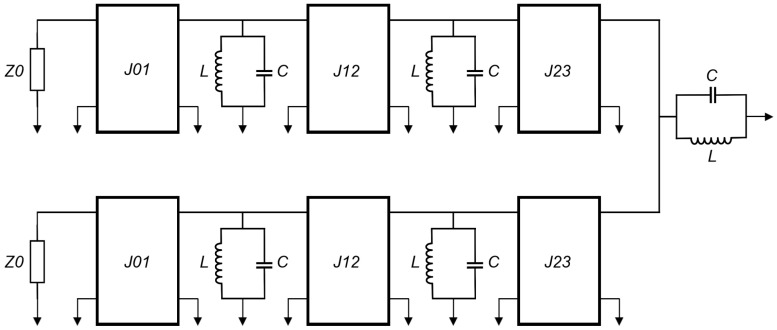
Fifth-order BPF circuit model with equal LC resonators and admittance inverters (*J*_01_ = 0.02, *J*_12_ = 0.0168, *J*_23_ = 0.0124, *C* = 14.0548 pF, *L* = 0.3724 nH).

**Figure 4:**
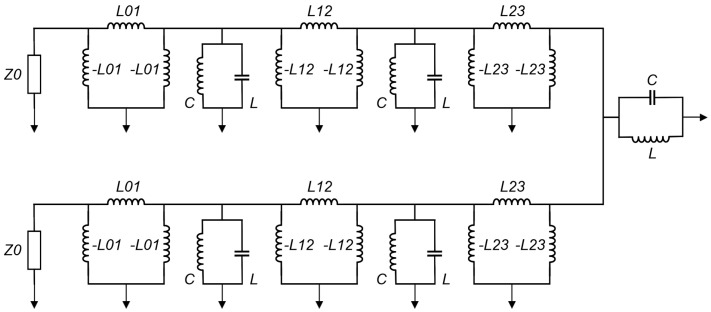
Fifth–order BPF circuit model, with equal LC resonators and inductor-only networks replacing J-inverters (*L*_01_ = 3.6172 nH, *L*_12_ = 4.2989 nH, *L*_23_ = 5.8542 nH).

**Figure 5:**
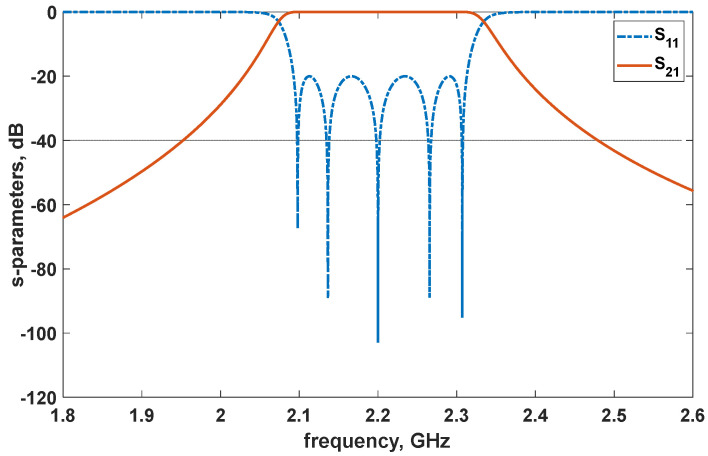
Fifth–order bandpass filter circuit model simulation results.

Admittance inverters (also known as J-inverters) are used to further transform the filter circuit model by converting all the series LC components (that is, L_2_C_2_ and L_4_C_4_ in this case) to parallel LC elements. This conversion gives rise to a BPF circuit model with equal shunt-only LC resonators and admittance inverters (that is, J-inverters), as demonstrated in Figure 3. The J-inverter values are determined using Equation (3) [1]. The J-inverters in Figure 3 are then replaced with inductor-only networks, as demonstrated in Figure 4. This relies on the transformation procedure reported in [1]. The J-inverter transformation makes it possible for the circuit model to be simulated in the circuit simulator of advanced design software (ADS), and the simulation results are described in Figure 5. The inductance values of the inductor-only networks are determined using Equation (4).

(3) J01=g0Z0 ; Jd,d+1=g12gdgd+1Z0 ; d=1,2,3,…

(4) Li,i+1=1Ji,i+1ω0 ; i=0,1,2,…

## 3. Microstrip Filter Design

This section reports on the fifth-order filter implementation using the proposed microstrip folded-arms square open-loop resonators. The section is divided into two subsections (resonator dimension and coupling coefficient and external quality factor). Each subsection discusses a stage in the microstrip filter design in detail and captures the sub-circuit parameters that are later put together to achieve the complete bandpass filter circuit results. 

### 3.1. Resonator Dimension

**Figure 6:**
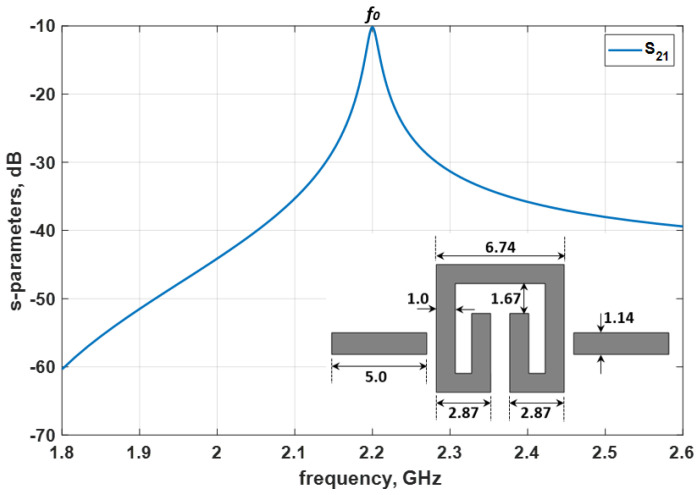
Practical dimensions of the proposed folded-arms square open–loop resonator at 2.2 GHz and the EM simulation response (all dimensions in mm).

The folded-arms square open-loop resonator (FASOLR) is constructed to resonate at the proposed bandpass filter center frequency, *f*_0_, of 2.2 GHz. The substrate material employed in the design is the Rogers RT/Duroid 6010LM with a relative permittivity, *ɛ_r_*, of 10.7, a loss tangent, tan *δ*, of 0.0023, and a substrate thickness, *h*, of 1.27 mm. The microstrip dimensions, the width (*w*) and the guided-wavelength (*λ_g_*), are determined from [1] by means of Equations (5) and (6), respectively. The substrate material’s effective permittivity or effective dielectric constant in Equation (6) is represented by *ɛ_eff_*, while the speed of light, *c*_0_, is a constant with a value of 3 × 10^8^ m/s. Using the technique reported in [1], the practical FASOLR dimensions were determined as shown in Figure 6.

(5) $$w=\frac{8h\;e^{A}}{e^{2A}-2}\;;\;A=\frac{Z_{0}}{60}\sqrt{\frac{\varepsilon_{r}+1}{2}}+\frac{\varepsilon_{r}-1}{\varepsilon_{r}+1}\left[0.23+\frac{0.11}{\varepsilon_{r}}\right]$$

(6) $$\lambda_{g}=\frac{c_{0}}{f_{0}\sqrt{\varepsilon_{eff}}}\;;\;\varepsilon_{eff}=\frac{\varepsilon_{r}+1}{2}+\frac{\varepsilon_{r}-1}{2}{\left(1+12\frac{h}{w}\right)}^{-0.5}$$

### 3.2. Coupling Coefficient and External Quality Factor Extraction

**Figure 7:**
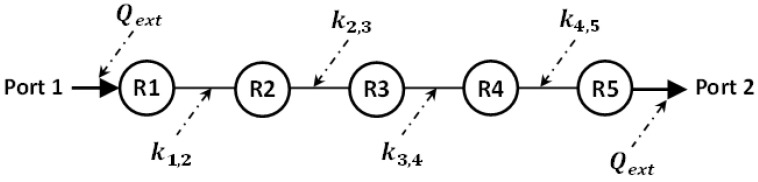
Fifth-order bandpass filter coupling coefficients and external quality factor arrangement (*k*_1,2_ = *k*_4,5_ = 0.087, *k*_2,3_ = *k*_3,4_ = 0.064, *Q_ext_* = 9.714).

**Figure 8:**
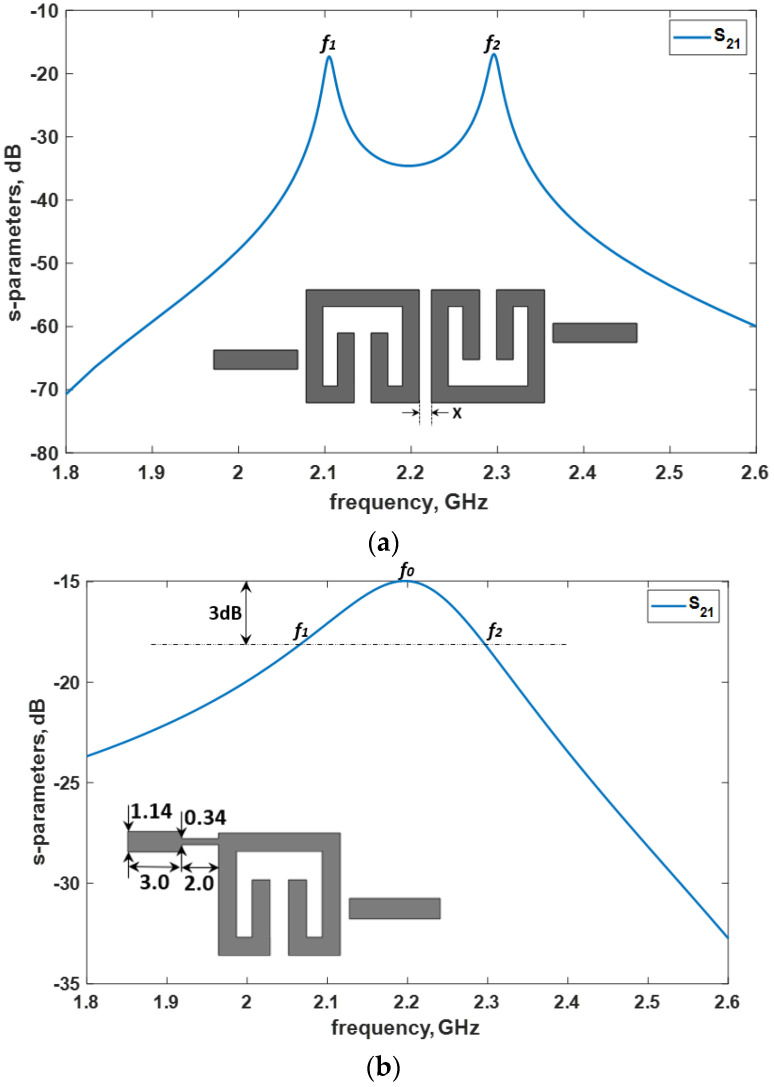
Microstrip layouts and EM simulation responses: (**a**) Coupling coefficient determination; (**b**) External quality factor determination.

The arrangement for the fifth-order bandpass filter theoretical coupling coefficients (*k*_1,2_, *k*_2,3_, *k*_3,4_, *k*_4,5_) between adjacent resonators (R1, R2, R3, R4, R5) is shown in Figure 7, alongside the theoretical external quality factor (*Q_ext_*) between each port and the corresponding adjacent resonator. The theoretical coupling coefficient values are calculated using Equation (7) [1], while the theoretical external quality factor value is determined based on Equation (8) [1]. The practical filter coupling coefficients and the practical *Q_ext_* are determined using Equations (9) and (10), respectively, as well as the detailed techniques reported in [1]. The resonator layouts and the electromagnetic (EM) simulation responses for obtaining the practical coupling coefficients, *k*, and the practical *Q_ext_* are shown in Figure 8.

(7) kd,d+1=FBWgdgd+1 ; d=1,2,3, 2…

(8) Qext=g0g1FBW

(9) k=(f22−f12)(f22+f12)

(10) Qext=f0(f2−f1)

## 4. Filter Simulation

**Figure 9:**
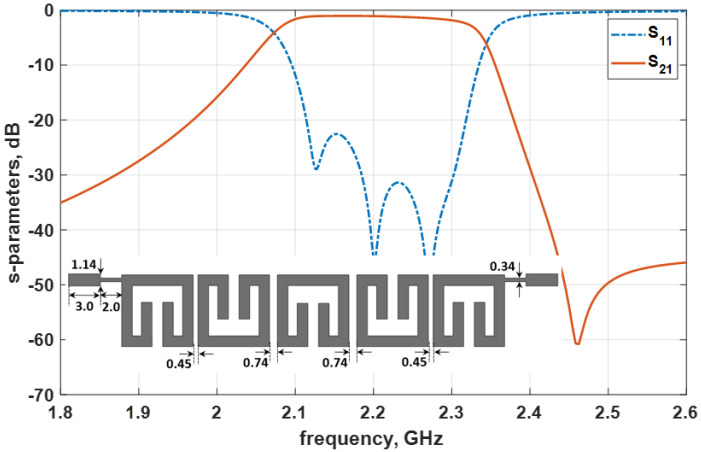
Fifth–order bandpass filter layout and the simulation responses for the folded-arms square open-loop resonator (all dimensions in mm).

The proposed filter layout using the folded-arms square open-loop resonator is developed following the assembly in Figure 7. The bandpass filter layout electromagnetic (EM) simulation is conducted on the Keysight Technologies USA, full-wave ADS EM simulator, with the filter layout and simulation responses presented in Figure 9. Looking at the simulation responses, the filter transmits at the expected centre frequency of 2.2 GHz, as expected. The EM simulation return loss is better that 22.6 dB, with a minimum insertion loss of 0.8 dB. The EM simulation uses a copper conductor with a cladding thickness of 35 micron (µm) and conductivity of 5.8 × 10^7^ S/m for the microstrip metals (top and bottom layers). The EM simulation did not consider the thickness variation of the filter substrate material and metal surface layer roughness.

## 5. Filter Experimentation

**Figure 10:**
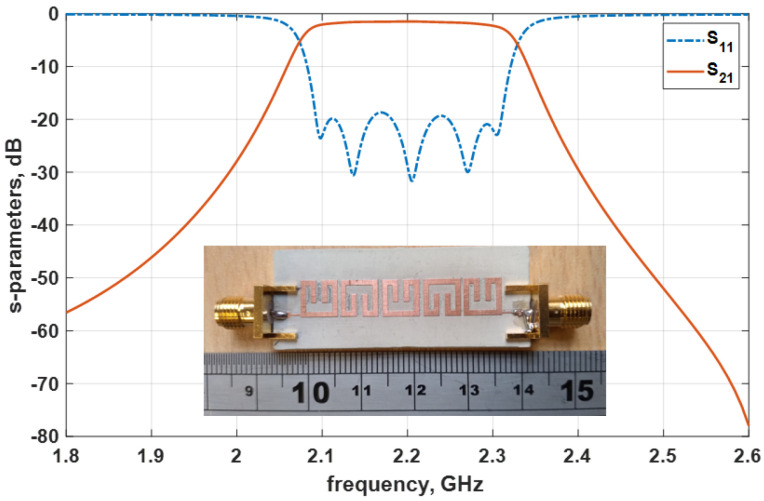
Image of the practical fifth–order filter and the measurement results.

The radio frequency PCB milling procedure is employed in the manufacture of the proposed bandpass filter. The substrate material employed for the filter circuit construction is Rogers RT/Duroid 6010LM with a relative permittivity, *ɛr*, of 10.8, a loss tangent, tan δ, of 0.0023, and a substrate thickness, h, of 1.27 mm. The images of the filter device and the measurement results are shown in Figure 10. The entire footprint of the constructed filter device is 36.08 mm by 6.74 mm, which corresponds to 0.26 λg × 0.05 λg, with λg representing the guided wavelength of the 50 Ω microstrip line with the centre frequency of 2.2 GHz. The filter measurement was conducted using a Keysight Technologies USA, N5230A Vector Network Analyzer (VNA), and two 50 Ω sub-miniature version A (SMA) connectors from RS Pro London UK, connected to each input/output port. The measurement results indicate a minimum insertion loss of 0.9 dB and a return loss better than 19.2 dB, as shown in Figure 10.

## 6. Results Discussion

**Figure 11:**
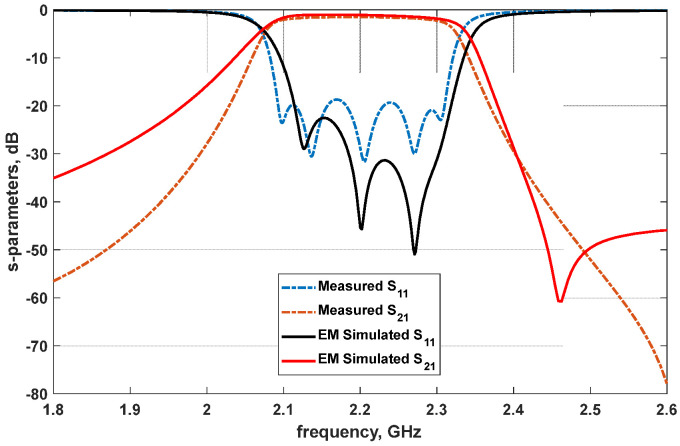
Comparison between the proposed filter model, EM simulation, and measurement results.

The proposed fifth-order bandpass filter EM simulation results and practical filter measurement results are jointly captured in Figure 11 for clear evaluation. The performance comparison of the designed fifth-order bandpass filter, when contrasted with the current state-of-the-art reported in some recently published related research, is presented in Table 1. Based on the comparison in Table 1, the designed filter is of small size as its overall footprint is relatively smaller than those reported in the other papers chosen for the comparison. The practical filter performance also shows that it is relatively less susceptible to both the loss factors compared (that is, the insertion and return loss factors). Looking at Figure 11, it can be seen that the simulation and measurement results are closely matched. However, though the measurement return loss is about 19.2 dB, the simulation return loss is better than 20 dB. Both the simulation and the measurement insertion losses are approximately 1.0 dB, with a slightly better simulation insertion loss of 0.9 dB. The simulated filter roll-off is slightly sharper than the measured filter roll-off, as shown in Figure 11. 

## 7. Conclusions

A fifth-order (that is, 5-pole) bandpass filter, based on the compact microstrip folded-arms square open-loop resonator, has been proposed, analysed, designed, implemented, fabricated, and measured. The filter device has been characterised using a Vector Network Analyzer, with the experimental responses used to validate the simulation responses. The presented measurement and EM simulation results indicate decent agreement. The EM simulation responses achieved a minimum insertion loss of 0.8 dB and a very good channel return loss of 22.6 dB. The measurement results, on the other hand, show a minimum insertion loss of 0.9 dB insertion loss and return loss of better than 19.2 dB. The designed filter component footprint is 36.08 mm by 6.74 mm (i.e., 0.26 λg × 0.05 λg), and λg is the guided wavelength of the 50 Ohm microstrip line at the centre frequency of the proposed fifth-order bandpass filter. This type of filter has popular applications in wireless transmitters and receivers. Its main function in the transmitter is to limit the bandwidth of the output signal to the band assigned for the transmission. By this effect, the transmitter is prevented from interfering with other stations. In the receiver, a bandpass filter permits signals within a certain band of frequencies to be received and decoded, while stopping signals at undesirable frequencies from getting through. The bandpass filter, reported in this paper, achieved an additional 20% physical device size reduction when compared to filters implemented using the conventional microstrip square open-loop resonator. The size reduction is due to the folded-arms of the FASOLR. The proposed compact filter would be very essential in radiofrequency (RF) front end of cellular radio base station transceivers as it would ensure the overall miniaturization of an entire system. Also, considering the fact that filters are used as building blocks for ubiquitous microwave components including multiplexers (e.g., diplexers), filtering antennas, filtering power dividers/combiners, etc., one can see the benefit of striving to make the filter design as compact as possible. Therefore, our proposed filter design is of huge benefit in terms of footprint, being of great interest to RF and microwave engineers and technicians alike. Practical technologies that would impact directly by the compactness of the proposed filter design technique include wireless communication systems. Take space crafts for example, and it may be seen that compact filters are employed in the design of diplexers used in the satellite communication systems of space crafts. This is because space crafts are built to an effective reduced mass and volume, as highlighted in [3]. Other receiver applications of the proposed filter include radar, drones, Wi-Fi, etc. All these technologies need filters for appropriate frequency selection.

## Figures and Tables

**Table 1:** Performances comparison between the designed filter and related recent publications.

Ref.	*f*_0_ (GHz)	Filter Order	Footprint (λ_g_ × λ_g_)	IL ^1^ (dB)	RL ^2^ (dB)
[16] [17] [19] [20]	3.5 4.5 3.4 3.0	3 2 5 4	0.17 × 0.16 0.28 × 0.09 0.37 × 0.10 0.24 × 0.07	0.6 1.6 0.5 1.0	13.0 10.0 10.0 11.0
This work	2.2	5	0.26 × 0.05	0.9	19.2

^1.^ insertion loss, ^2.^ return loss.

## References

[1] Hong J.-S. (2011). Microstrip Filters for RF/Microwave Applications.

[2] Prasetya B., Rohmah Y.S., Nurmantris D.A., Mulyawati S., Dipayana R. (2021). Band pass filter comparison of hairpin line and square open-loop resonator method for digital TV community. Bull. Electr. Eng. Inform..

[3] Nwajana A.O., Yeo K.S.K. (2016). Microwave diplexer purely based on direct synchronous and asynchronous coupling. Radioengineering.

[4] Mitic M., Nedelchev M., Kolev A., Marinkovic Z. (2020). ANN based design of microstrip square open loop resonator filters. Proceedings of the 2020 Joint International Conference on Digital Arts, Media and Technology with ECTI Northern Section Conference on Electrical, Electronics, Computer and Telecommunications Engineering (ECTI DAMT & NCON), Pattaya, Thailand, 11–14 March 2020.

[5] Astuti D.W., Perkasa R.A., Muslim, Pahlevi T.A. (2019). HMSIW bandpass filter using square open loop resonator for short range device applications. Proceedings of the 2019 IEEE 14th Malaysia International Conference on Communication (MICC), Selangor, Malaysia, 2–4 December 2019.

[6] Nwajana A.O., Yeo K.S.K. (2016). Multi-coupled resonator microwave diplexer with high isolation. Proceedings of the 46th European Microwave Conference (EuMC), London, UK, 4–6 October 2016.

[7] Shaheen M.M., Mahmoud N.M., Ali M.A., Nasr M.E., Hussein A.H. (2022). Implementation of a highly selective microstrip diplexer with low insertion loss using square open-loop resonators and a T-junction combiner. Radioengineering.

[8] Jarchi S., Khalily M., Tafazolli R. (2020). Effects of metamaterial loading on miniaturization of loop and open loop microstrip filters. Proceedings of the 2020 International Conference on UK-China Emerging Technologies (UCET), Glasgow, UK, 20–21 August 2020.

[9] Astuti D.W., Juwanto, Alaydrus M. (2013). A bandpass filter based on square open loop resonators at 2.45 GHz. Proceedings of the 3rd International Conference on Instrumentation, Communications, Information Technology and Biomedical Engineering (ICICI-BME), Bandung, Indonesia, 7–8 November 2013.

[10] Santana M.P., Barbosa E.V.S., Rondineau S.R.M.J. (2020). Design of Ku–band filters and diplexer for a heterodyne transceiver using coupled open–loop resonator cells. Proceedings of the 2020 Workshop on Communication Networks and Power Systems (WCNPS), Brasilia, Brazil, 12–13 November 2020.

[11] Gorur A.K., Turkeli A., Dogan E., Karpuz C., Gorur A. (2021). A novel compact microstrip diplexer with closely spaced channels. Proceedings of the 2020 50th European Microwave Conference (EuMC), Utrecht, the Netherlands, 12–14 January 2021.

[12] Mansour G., Rahim M.K.A., Aldeeb H.A. (2021). Cross–Coupled microstrip bandpass filters with finite frequency transmission zeros. Proceedings of the 2021 IEEE 1st International Maghreb Meeting of the Conference on Sciences and Techniques of Automatic Control and Computer Engineering MI-STA, Tripoli, Libya, 25–27 May 2021.

[13] Alkhafaji M.K. (2020). Electrically coupled folded arm resonator with the feedline patch antenna for spurious harmonic suppression and bandwidth improvement. Iraqi J. Electr. Electron. Eng..

[14] Ledezma L.M. (2011). A Study on the Miniaturization of Microstrip Square Open-Loop Resonators. Master of Science Thesis.

[15] Cameron R.J., Kudsia C.M., Mansour R.R. (2018). Microwave Filters for Communication Systems: Fundamentals, Design, and Applications.

[16] Hong J.-S., Lancaster M.J. (1996). Coupling of microstrip square open-loop resonators for cross-coupled planar microwave filters. IEEE Trans. Microw. Theory Tech..

[17] Sanchez-Martinez J.J., Marquez-Segura E., Lucyszyn S. (2014). Design of compact wideband bandpass filters based on multiconductor transmission lines with interconnected alternate lines. IEEE Microw. Wirel. Compon. Lett..

[18] Zhang P., Liu L., Chen D., Weng M.-H., Yang R.-Y. (2020). Application of a stub-loaded square ring resonator for wideband bandpass filter design. Electronics.

[19] Nwajana A.O., Obi E.R. (2022). A review on SIW and its applications to microwave components. Electronics.

[20] Zhang R., Zhu L. (2012). Synthesis design of a wideband bandpass filter with inductive coupling short-circuited multi-mode resonator. IEEE Microw. Wirel. Compon. Lett..

[21] Shaman H.N. (2012). New S-band bandpass filter (BPF) with wideband passband for wireless communication systems. IEEE Microw. Wirel. Compon. Lett..

[22] Ayinala K.D., Sahu P.K. (2022). An SRR–loaded compact triple–band 4–Element MIMO design for WLAN/WiMAX/C–band applications. Proceedings of the 2022 IEEE Wireless Antenna and Microwave Symposium (WAMS), Rourkela, India, 5–8 June 2022.

[23] Nedelchev M., Mitic M., Kolev A., Marinkovic Z. (2020). Modeling and design of microstrip coupled resonator filters based on ANNs. Proceedings of the 2020 43rd International Conference on Telecommunications and Signal Processing (TSP), Milan, Italy, 7–9 July 2020.

